# Herbal remedies used by traditional healers to treat haemorrhoids in Tabora region, Tanzania

**DOI:** 10.1080/13880209.2022.2136204

**Published:** 2022-10-28

**Authors:** David Sylvester Kacholi, Halima Mvungi Amir

**Affiliations:** Department of Biological Sciences, Dar es Salaam University College of Education, University of Dar es Salaam, Dar es Salaam, Tanzania

**Keywords:** Ethnomedicine, herbalists, piles, Sikonge, traditional remedies, Urambo

## Abstract

**Context:**

Haemorrhoids are one of the most common gastrointestinal disorders in humans. In Tanzania, particularly in the Tabora region, medicinal plants (MPs) are used by traditional healers (THs) to treat haemorrhoids, but no study has explicitly attempted to compile these treatments.

**Objective:**

This study documents MPs used by THs of the Tabora region in Tanzania to treat haemorrhoids.

**Materials and methods:**

A semi-structured questionnaire was used to gather ethnobotanical data from 44 THs on MPs used to treat haemorrhoids, parts used, preparation methods and administration routes. The collected ethnobotanical data were analysed by computing percentage frequencies and relative frequency citations.

**Results:**

Twenty-six MPs belonging to 19 families and 25 genera, used to manage haemorrhoids, were documented. Fabaceae was the dominant family (four species), whereas shrubs constituted a high proportion (38.46%) of the MPs, and the root was the most (30.3%) utilized plant part. Decoction (38.5%) and topical application (53.8%) were the most preferred preparation and administration techniques. Most MP materials (76.9%) were sourced from the wild. *Aloe vera* (L.) Burm.f. (Asphodelaceae) (68%), followed by *Allium sativum* L. (Alliaceae) (66%) and *Psidium guajava* L. (Myrtaceae) (66%) were the most utilized MPs. Among the recorded MPs, 12 are reported for the first time for the treatment of haemorrhoids. The recorded MPs are believed to possess anti-inflammatory properties that aid in managing inflammation associated with bowel diseases, including haemorrhoids.

**Conclusions:**

This study has documented valuable MPs used to manage haemorrhoids and provides a basis for further studies to discover efficient and affordable anti-haemorrhoid drugs.

## Introduction

Haemorrhoids, also called piles, are swollen veins in the anus and lower rectum. The ailment is mainly caused by increased pressure in the veins due to straining when trying to have a bowel movement, or any activity that causes strain. As pressure increases, blood pools in the veins increase, which causes them to swell, thus stretching the surrounding tissue (Peery et al. 2015). The most common and severe effects of haemorrhoids are perianal thrombosis, and incarcerated prolapsed internal haemorrhoids with subsequent thrombosis (Carey and Whelton 2018). The disease is considered a significant cause of morbidity and has economic as well as social impacts on society (Riss et al. 2012). The economic impact includes a burden on health systems and loss of working days, while social impact is linked to lifestyles, for instance, interpersonal, food and hygiene, and sexual habits (Rubbini and Ascanelli 2019). Moreover, the disease causes physical and psychological distress (Khan et al. 2015), and considerably affects the patient’s quality of life due to bleeding occurring with or without defaecation, anal pain, and itching (Guttenplan 2017).

Various studies revealed that constipation, obesity (Peery et al. 2015; Kibret et al. 2021), diarrhoea, insufficient dietary fibre, chronic straining during defaecation (Everhart and Ruhl 2009), abdominal obesity, depression, pregnancy (Lee et al. 2014), hypertension, smoking (Hong et al. 2022), as well as advancement in age and sedentary lifestyle (Khan et al. 2015), are common risk factors for the development of haemorrhoids. Globally, very few studies have been conducted to study the prevalence of haemorrhoids. For instance, in Africa, the prevalence of the disease is 18% in Egypt (ElBatea et al. 2011), and 13.1% in Ethiopia (Kibret et al. 2021), while in Tanzania, no study has attempted to study the prevalence and allied risk factors of haemorrhoids.

In Africa, the dependence on MPs is due to the scarcity of modern health facilities, and the incapacity to pay for contemporary remedies and health services by rural residents. Likewise, the popularity of herbal remedies is due to the locals’ high spiritual and cultural acceptability (Maroyi 2013; Mathibela et al. 2019). The indigenous knowledge of MPs against various illnesses is well accrued in areas where the use of the plants is yet paramount. In Tanzania, MPs play a vital role in primary health care for most of the rural population. However, MPs are at risk in the country due to various factors such as debarking, uprooting and branch cutting (Mbinile et al. 2020), as well as deforestation, degradation, expansion of agriculture, climate change, habitat loss, urbanization, and the use of plants for firewood and charcoal (Kacholi and Mvungi 2021). Additionally, traditional knowledge is greatly threatened due to the lack of a written record (Kacholi and Amir 2022) and conservative inheritance patterns (Hu et al. 2020). The knowledge is well stored in the elderly, while the young generation shows no interest in this healing science as they prefer to look for higher-income occupations in urban areas (Tahir et al. 2021).

Thus, considering the weak traditional recording and knowledge transfer system, as well as alarming rates of environmental degradation, documenting MPs and their ethnobotanical information is an essential task. In the selected study areas, Sikonge and Urambo districts of the Tabora region, 94.8% and 82.3% of the people, respectively, live in rural areas where there are limited modern health facilities; hence, there is high reliance on traditional healers (THs) for their primary healthcare needs. Among the risk factors for the development of haemorrhoids in the study area is diarrhoea; the disease has a prevalence of 5.1%, which is relatively low compared to other regions in the country but is the fourth for causing morbidity in the study areas (Pual and Muluken 2019). Besides, no study has endeavoured to explicitly compile MPs used for treating haemorrhoids in Tanzania. Therefore, the objective of this study was to document THs ethnobotanical knowledge related to the treatment of haemorrhoids in the Tabora region, Tanzania.

## Materials and methods

### Study area

**Figure 1.**
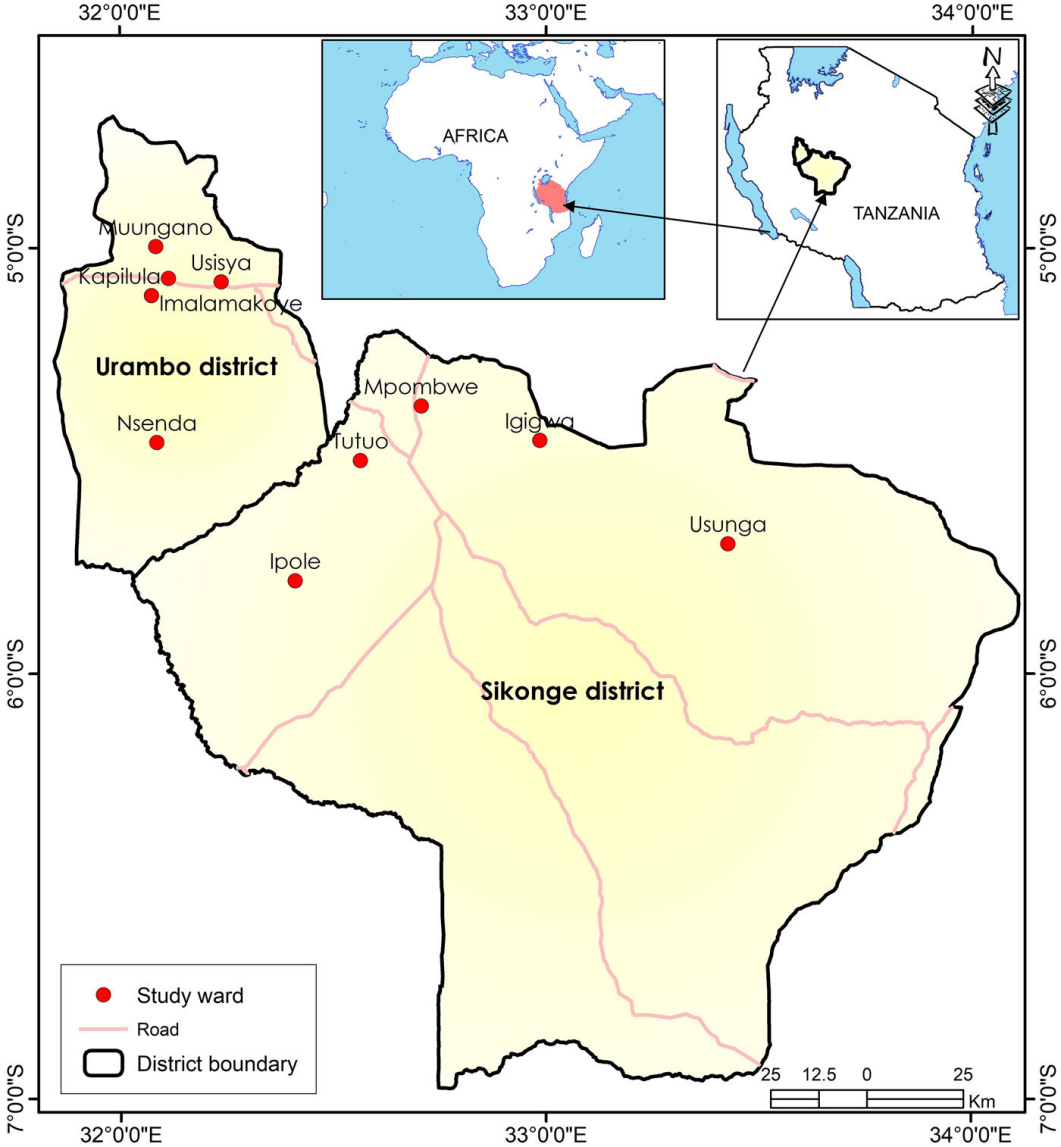
Localities of the study sites in the two districts, setting of the districts in the country and location of the country in Africa.

This study was carried out in two districts, namely, Urambo district (latitudes of 04°41′ to 05°44′S and longitudes 31°51′ to 32°26′E) and Sikonge district (latitudes 05°15′ to 06°45′S and longitudes 32°15′ to 33°45′E) located to the western and southern side of Tabora region, respectively (Figure 1). A total of 10 wards, five from Urambo district (Nsenda, Imalamakoye, Usisya, Kapilula and Muungano), and the other five from Sikonge district (Ipole, Tutuo, Mpombwe, Igigwa and Usunga) were involved. Generally, the two districts cover 43.7% of the region, and constitute 17.4% of the regional population (Table 1). The two districts are inhabited by the Nyamwezi tribe, whose crop cultivation and livestock rearing production systems are the predominant agricultural activities. Major cash and food crops grown in the areas are tobacco and rice, respectively. In Tanzania, the Nyamwezi are famous for their traditional healing practices (Augustino et al. 2011; Kacholi and Mvungi 2021); hence, the selection of the districts to document their knowledge is related to haemorrhoid treatment.

**Table 1.** Population, area and density of the study areas.

District	Population	Area (km^2^)	Population density (no./km^2^)
Urambo	192,781	5416	35.60
Sikonge	179,883	27,873	6.84

### Data collection

The survey was conducted between August and October 2020. A semi-structured questionnaire, field tour and personal interviews with THs were based on the standard ethnobotanical approaches (Cunningham 2001). The interview was administered in the Swahili language. Forty-four THs (21 from Urambo and 23 from Sikonge districts) were involved in the study. During the interview, information on MPs used for curing haemorrhoids, plant parts used, the form in which it is used, preparation and administration methods, and threats to MPs knowledge and use were gathered. On-the-spot proof of identity of acquainted plant species was done during the field tour. Some plants not identified during the tour were collected for further identification and confirmation in the DUCE herbarium. Only those confirmed by at least three THs were reported in this study. Botanical names were confirmed using the International Plant Name Index (IPNI).

### Research clearance and ethical consent

Before starting with data collection, research clearance was obtained from the office of the Vice-Chancellor of the University of Dar es Salaam. Voluntarily verbal consent of all 44 involved THs was requested before interviewing them. All THs were assured that the information obtained from this study would be used for academic purposes only.

### Data analysis

The information was analysed with descriptive statistics using Microsoft Office Excel version 2013 (Redmond, WA). The relative frequency citation (RFC) for each medicinal plant (MP) was calculated to determine the number of THs that considered a particular plant species as being worth anti-haemorrhoidal. RFC values range between 0 and 1, whereby RFC of 1 suggests the uppermost level of THs consensus on using a particular plant species as antidiarrheal. The RCF was calculated using the following formula:

$$\mathrm{RFC}=\;\frac{\mathrm{FCs}}{N}=\;\sum_{i=1}^{N}\frac{\mathrm{uRi}}{N}$$

where FC is the number of THs who cited a particular plant species and N is the total number of THs involved in the survey.

## Results and discussion

### Medicinal plant diversity

Twenty-six species belonging to 19 families and 25 genera were documented as used by THs as anti-haemorrhoidal (Table 2). Fabaceae was the dominant family with four species (15.4%), followed by Amaryllidaceae, Euphorbiaceae and Combretaceae, with two species each (7.7%). The remaining families were represented by only one species each. The Fabaceae was also the most reported family used for haemorrhoidal treatment in Nigeria (Ariyo et al. 2020). Similarly, the family Fabaceae is reported to be dominant in other ethnobotanical studies conducted in South Africa (Maema et al. 2019), Zimbabwe (Maroyi 2013) and Ethiopia (Tahir et al. 2021). Globally, the Fabaceae is the third-largest plant family in terms of species richness after Orchidaceae and Asteraceae (Singh et al. 2021), and it has a higher adaptation potential over a wider range of habitats (Bareke 2018). For instance, in Tanzania, the Fabaceae dominate 37% of the Morogoro region (Kacholi et al. 2015) and 26–38% of the coastal forests (Kacholi 2019; Ligate et al. 2019).

**Table 2.** List of medicinal plants used by traditional healers in treating haemorrhoids in Tabora region.

Family and scientific name	Local name	Life forms	Source	Part used	RFC	Preparation and administration	Literature reports supporting the claim
Amaryllidaceae							
*Allium cepa* L.	Kitunguu maji	H	Cu	Bu	0.45	Heated, crushed and applied externally to the affected part daily	Shettar et al. (2021)
*Allium sativum* L.	Kitunguu saumu	H	Cu	Bu	0.66	Crush to make a juice, mix with sugar/honey and ingest a half tablespoon thrice per day, or dry bulb and apply to the affected part	Ariyo et al. (2020), Soladoye et al. (2010), and Thiyam et al. (2014)
Anacardiaceae							
*Ozoroa reticulata* (Baker f.) R. Fern. & A. Fern.	Mukalakala	S	Wi	B	0.50	Boil in water, then drink the decoction, a teaspoon twice a day	Not found
Annonaceae							
*Annona senegalensis* Pers.	Mutopetope	S	Wi	B	0.07	Extract, make ropes and wear	Not found
Apocynaceae							
*Strophanthus eminii* Asch. ex Pax	Musungululu	S	Wi	R	0.09	Boil chopped roots and then take decoction orally	Not found
Boraginaceae							
*Ehretia amoena* Klotzsch.	Mukirika	S	Wi	B	0.47	Boil to make a decoction and drink a cup twice a day	Shukla and Kaur (2018)
Combretaceae							
*Combretum zeyheri* Sond.	Musana	T	Wi	R, B	0.61	Boil to make the decoction, and drink a cup of it three times a day	Augustino et al. (2011)
*Terminalia sericea* Burch. Ex. DC.	Muzima	T	Wi	R, L	0.56	Boil in water and drink decoction three-times a day	Augustino et al. (2011)
Cyperaceae							
*Cyperus articulatus* L.	Mundagolago	H	Wi	Wh	0.13	Crush the plant and massage the anus gently daily	Not found
Euphorbiaceae							
*Jatropha gossypiifolia* L.	Mubono	S	Wi	R, L	0.38	Boil to make a decoction and then drink it in a cup twice a day	Ariyo et al. (2020) and Soladoye et al. (2010)
*Euphorbia tirucalli* L.	Munyaa	S	Wi	R	0.41	Pound and mix in tea. Apply to the affected part twice daily.	Osman et al. (2020)
Fabaceae							
*Pterocarpus tinctorius* Welw.	Mukulungu	T	Wi	B	0.41	Boil in water and apply Sitz bath twice a day	Not found
*Erythrina abyssinica* Lam. ex DC.	Mulinzi	T	Wi	B		Boil in water to make a decoction and take it orally	Not found
*Dichrostachys cinerea* (L.) Wight & Arn.	Mutundulu	S	Wi	L, R, B	0.32	Boil to make a decoction and drink a cup twice a day	Not found
*Grewia bicolor* Juss.	Mukoma	S	Wi	B	0.07	Extract, make ropes and wear	Not found
Lamiaceae							
*Plectranthus argentatus* (S.T.Blake) P.I.Forst. & T.C.Wilson	Mulavumba	H	Wi	L	0.27	Burn and then massage anus gently, once daily	Not found
Liliaceae							
*Aloe vera* (L.) Burm.f.	Mulovera	Su	Cu	Wh	0.68	Peel and apply to the anus by massaging the affected part	Shettar et al. (2021) and Soladoye et al. (2010)
Moringaceae							
*Moringa oleifera* Lam.	Mulonge	T	Cu	L	0.54	Crush mix with hot water and use for Sitz bath	Soladoye et al. (2010)
Myrtaceae							
*Psidium guajava* L.	Mupera	T	Cu	L	0.66	Crush and apply the affected part by massaging	Parvaiz et al. (2013) and Soladoye et al. (2010)
Pedaliaceae							
*Sesamum angolense* Welw.	Mulendawima	H	Wi	R	0.20	Crush and apply the affected part by massaging	Not found
Phyllanthaceae							
*Phyllanthus engleri* Pax	Mung’ongo Ntandala	S	Wi	B	0.45	Boil in water and use for Sitz bath	Not found
*Hymenocardia acida* Tul.	Mupala	S	Wi	R, L	0.59	Crush, soak in water and drink the solution three-times a day	Sofidiya et al. (2009)
Rubiaceae							
*Crossopterix febrifuga* (Afzel. ex G.Don) Benth.	Musasambeke	T	Wi	R	0.09	Pound and mix in tea or porridge, then drink	Not found
Rutaceae							
*Zanthoxylum chalybeum* Engl.	Mulungulungu	T	Wi	R	0.16	Dry and pound the roots, then soak in water and drink	Tuasha et al. (2018)
Solanaceae							
*Solanum incanum* L.	Ntalantu	H	Cu	F, L	0.18	Crush and massage affected part of the rectum	Ghazanfar and Al-Al-Sabahi (1993)
Vitaceae							
*Cissus quadrangularis* L.	Vula-wo-nsuwi/Mutandamwaka	H	Wi	Wh	0.61	Crush and massage affected part of the rectum	Panthong et al. (2007)

H: herbs; T: trees; S: shrubs; R: roots; L: leaves; B: bark; F: fruits; Wh: whole plant; Bu: bulb; Su: succulents; Cu: cultivated land; Wi: wild.

The 26 plant species reported in this study are less than those reported being used for treating haemorrhoids in southwestern Nigeria (144 species) (Soladoye et al. 2010). However, the present study recorded more plant species compared to that recorded in the Gujrat District of Pakistan (22 species) (Parvaiz et al. 2013) and for Oyo State, Nigeria (25 species) (Ariyo et al. 2020). Among the recorded MPs in the present study, 14 species were reported elsewhere (Table 2), while the remaining 12 are reported in the present study for the first time as anti-haemorrhoidal. The difference in the MPs richness between the present study and the above-cited studies could be attributed to environmental factors such as geographic, biotic and abiotic variations, which tend to affect plant community diversity in different locations (Li et al. 2020). Also, the sample size involved in a study could be the source of the variations as it is expected that large sample size could lead to more information than a small sample size.

The five most-cited plant species in terms of RFC were *Aloe vera* (L.) Burm.f. (Asphodelaceae) (68%), followed by *Allium sativum* L. (Alliaceae) and *Psidium guajava* L. (Myrtaceae) with 66% each, *Combretum zeyheri* Sond. (Combretaceae) and *Cissus quadrangularis* L. (Vitaceae) (with 61% each), *Hymenocardia acida* Tul. (Phyllanthaceae) (59%) and *Terminalia sericea* Burch.ex DC. (Combretaceae) (56%) (Table 2). These species are believed to have anti-inflammatory properties that help in soothing the inflammation of the haemorrhoids. For instance, *A. vera* gel (Langmead et al. 2004), *A. sativum* (Arreola et al. 2015), *C. quadrangularis* (Panthong et al. 2007), *H. acida* (Sofidiya et al. 2009) and *T. sericea* (Mongalo et al. 2016) have been experimentally confirmed to have the anti-inflammatory effects that help in treating acute inflammatory ailments such as piles. Hence, these MPs can be used to develop efficient and affordable contemporary drugs against haemorrhoids that can be used widely in the country and elsewhere.

### Source of medicinal plants

Of the 26 recorded MPs, 76.9% were harvested from the communal wild areas, while 23.1% were from cultivated lands and home gardens. The same finding is reported in Dalle District, Ethiopia, whereby 90.4% of the herbal remedies are collected from the wild (Tuasha et al. 2018). Similarly, most MPs are accessed from the wild in Morogoro (Kacholi and Amir 2022) and Kagera region (Moshi et al. 2012) in Tanzania. The wild’s dependency is because it constitutes free resources and no permits are necessary for collection. Also, MPs users perceive cultivated plants as less effective than wild ones (Moeng and Potgieter 2011), and the absence of scientific evidence on the efficacy of cultivated MPs remains a limiting factor (Mangoale and Afolayan 2020). Thus, wild habitats are still crucial for the livelihood of the rural community in the region. Also, the finding indicates that the cultivation of MPs in the region is very limited, and it provides an early warning that the natural vegetation in the wild is open to overexploitation. Therefore, protecting and managing wild environments should be prioritized to ensure a sustainable supply of the MPs. Furthermore, this study recommends the *ex situ* cultivation of MPs as a conservation approach and an excellent means of reducing the overexploitation of wild resources. The strategy is vital in ensuring future access to herbal remedies to uphold the rural primary health care system and providing materials for new low-priced drug discoveries through modern science.

### Growth form analysis

The analysis of growth forms showed that shrubs accounted for 38.46% of the recorded MPs, followed by trees (30.77%), herbs (26.92%) and succulents (3.85%). A similar finding was reported in India (Thiyam et al. 2014), wherein shrubs constituted the highest proportion of MPs used in treating haemorrhoids. Also, the finding concurs with other ethnobotanical studies conducted in Ethiopia (Jima and Megersa 2018; Tuasha et al. 2018), which reported shrubs to constitute a large proportion of MPs used for the treatment of various human ailments. However, in Nigeria (Ariyo et al. 2020), trees were reported to be the most commonly used plant form for managing haemorrhoids. The prevalence of shrubs and trees in recipes indicates that the locals are conversant about using higher plants in treatments. The THs prefer the two growth forms as they are accessible throughout the year, hence, warranting sustainable management of the ailment (Kacholi and Amir 2022). Since MPs resources are harvested in increasing volumes from wild areas, there is a danger of extinction for rare and most utilized species (Chen et al. 2016). Therefore, measures relating to MPs’ conservation (*in situ* or *ex situ*) must be developed for a sustainable supply of MPs’ resources. Also, since the cultivation of MPs is limited by the lack of appropriate technologies and cultural beliefs (Kisangau and Herrmann 2007), there is a need to train THs to use inexpensive and appropriate propagation techniques to sustain the supply of the MPs.

### Plant parts used

**Figure 2.**
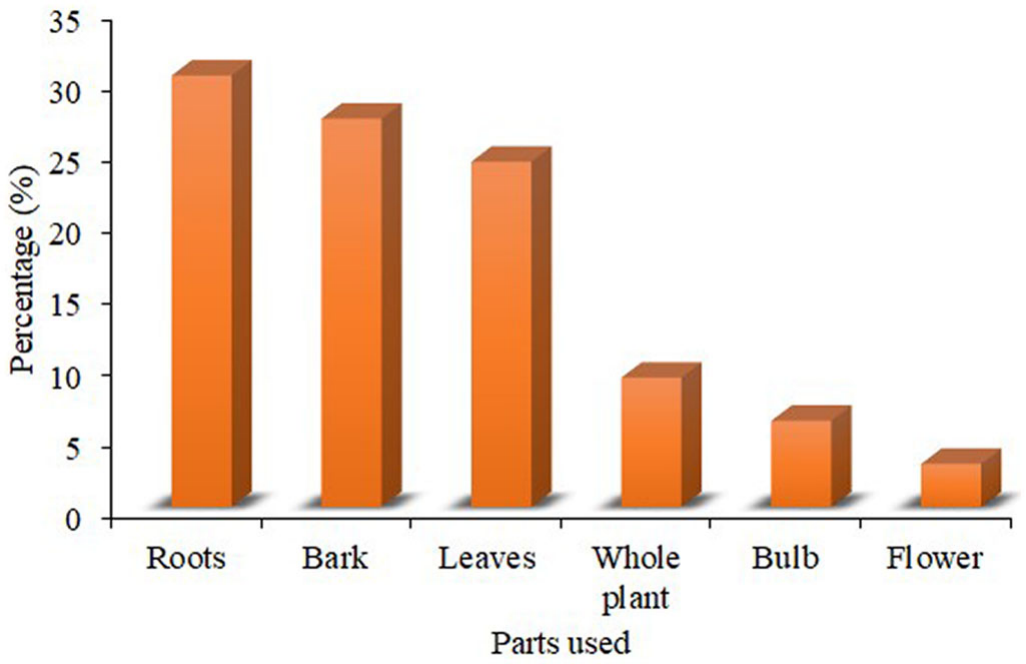
Medicinal plant parts used by THs to treat haemorrhoids.

The study revealed that THs harvest different plant parts, including roots, bark, leaves, fruits and whole plants, to prepare herbal remedies (Figure 2). These plant parts were used singly or in combination with other parts. The findings showed roots (30.3%) to be the most preferred used part, followed by bark (27.3%, nine species) and leaves (24.2%, eight species). Similar findings in China (Hu et al. 2020), Ethiopia (Jima and Megersa 2018; Tahir et al. 2021), South Africa (Mathibela et al. 2019) and Uganda (Oryema et al. 2021) reported roots to be the preferred part for making traditional remedies. The roots are highly preferred due to the possession of a high concentration of bioactive compounds and nutrients compared to other plant parts (Chinsembu 2016). However, harvesting roots and bark have detrimental impact on the plant’s survival (Cunningham 2001; Oryema et al. 2021). The harvest of roots has effects on plant anchorage, nitrogen fixation and root systems of the adjacent plants, while the uncontrolled harvest of bark, particularly of slow-growing species, can result in extinction (Kacholi and Mvungi 2021). Thus, using leaves as an alternative is usually advocated (Chen et al. 2016; Jena et al. 2017; Megersa et al. 2019) as a more sustainable practice due to their renewability and less detrimental effect on the parent plant.

### Preparation and administration of remedies

Most herbal remedies were prepared from fresh plant parts (85.2%), or dried form (14.8%). Water and other hot drinks such as tea, porridge and milk are used as the medium for preparing recipes for treating haemorrhoids. This finding agrees with studies conducted in Ethiopia (Tefera and Kim 2019; Tahir et al. 2021), wherein the use of freshly harvested plant parts are preferred over dry forms due to their higher efficacy in treatment. Usually, dry forms lose their effectiveness due to evaporation and deterioration of bioactive ingredients during the drying process (Chekole 2017).

**Figure 3.**
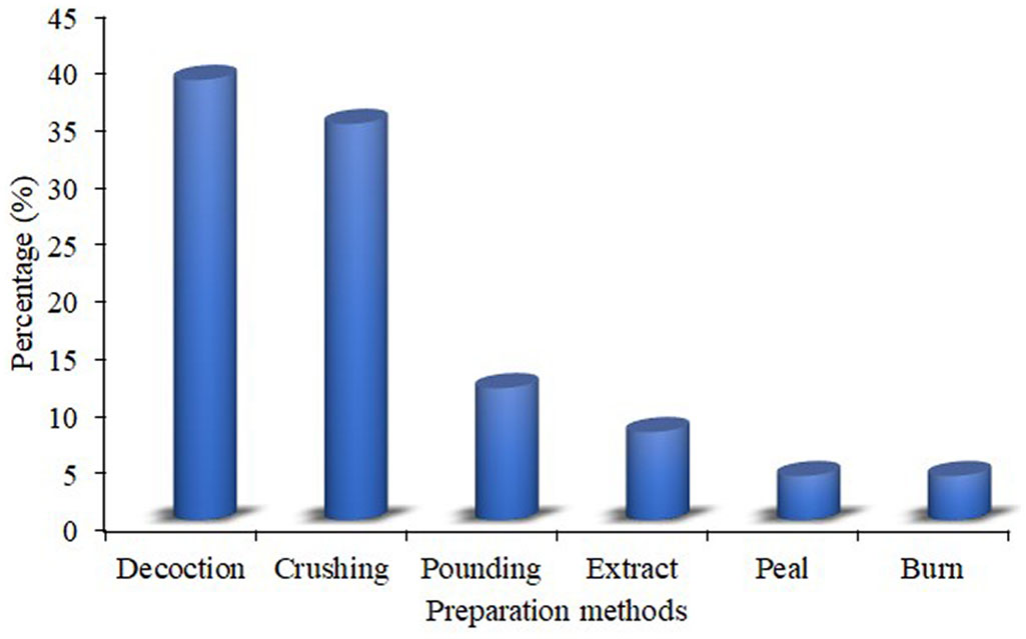
Modes of preparing herbal remedies.

Most of the remedies were prepared by decoction (38.5%), followed by crushing (34.6%), pounding (11.5%), with the remaining methods accounting for 15.4% of the total (Figure 3). Likewise, the findings reported in Ghana (Appiah et al. 2018) and China (Hu et al. 2020) indicated decoction to be the most used method in preparing remedies. The decoction method might be associated with speeding up the extraction of active ingredients from the materials, detoxifying poisonous compounds and sterilizing used materials (Maema et al. 2019). Moreover, the remedy preparations involve using different ingredients and solvents. For instance, of the recorded curative plants, 38.5% are prepared directly without adding any component, 46.2% of the materials are prepared using water, and the remaining 15.3% are mixed with tea, honey, milk, porridge or sugar. Water is preferred in the preparations as it is not expensive and a more efficient solvent that can extract more soluble metabolites than milk. Additives such as honey, sugar, milk and butter are commonly used to increase the efficacy and potency of the remedies (Wubetu et al. 2017), making the formulation palatable by adding flavour and taste, and avoiding abdominal discomfort (Eshete and Molla 2021).

The THs administer herbal remedies topically (53.8%) and orally (46.2%). Among the externally applied remedies, 78.6% are applied by massaging or creaming the infected part, while 21.4% are applied through a Sitz bath. The finding is in-line with those reported in Nigeria (Ariyo et al. 2020), whereby topical application and oral administration were the major routes of administering remedies in treating haemorrhoids. According to the THs, topical remedies are mainly preferred to treat external haemorrhoids because they reduce inflammation, stop bleeding, and relieve pains and itching more quickly than the oral route. Furthermore, remedies for patients with chronic haemorrhoids are commonly administered simultaneously through both routes to speed up recovery.

Regarding the dosage, THs used a tablespoon, cup, spoon, finger, fist or hand palm to determine the herbal dosage for treatment. The administration varied significantly from half or full cups of the recipe once or twice or three-times daily, and full or half of the tablespoon twice or three-times daily. The variation was determined by age, health history, physical fitness status, the duration of the disease, presence or absence of pregnancy, and other associated factors. In case of over-dosage, the THs recommend a cup of milk, which they believe minimizes likely side effects. However, as reported in other studies, the non-existence of accurate dosage and quality control mechanisms among the THs are significant drawbacks in traditional medicinal practices (Jima and Megersa 2018). The lack of accurate dosage and control mechanisms can lead to changes in the immune system, make the malady chronic to patients, or cause severe life-threatening health conditions to patients due to the toxicity of the remedies. Thus, knowing the appropriate dosage is imperative to THs to avoid lethal effects on users.

### Threats to medicinal plant knowledge and use

Agricultural expansion (45%), followed by deforestation (31%), fire (14%) and overgrazing (10%) were mentioned by THs to be the major threats to the MPs resources. Likewise, in Ethiopia, agricultural expansion is the main challenge in conserving MPs (Tefera and Kim 2019). Moreover, the THs established that the younger generations are unwilling to learn this traditional healing practice. Hence, a great deal of important information regarding MPs could be lost when THs and elders die without sharing knowledge with the younger generations. The THs recommended planting MPs, followed by soil and water conservation, provision of awareness and protection of nearby forests as suitable methods for conserving and protecting MPs. In addition, the study suggests that *in situ* and *ex situ* conservation, good agricultural practices, and sustainable harvesting solutions should be sufficiently considered for the sustainability of MPs resources.

## Conclusions

The study showed that the region is rich in abundance and diversity of MPs used to manage haemorrhoids and indigenous traditional knowledge on using the resources. The study revealed 26 MPs distributed among 19 families and 25 general; and Fabaceae was the most utilized family. Most of the reported MPs are trees obtained from the wild, and most remedies are prepared in the form of decoction. The most utilized plant part was the root. The reported MPs with the highest relative citation frequencies (RCFs) can be valuable sources for discovering efficient and affordable novel drugs. Therefore, further investigation is required on the therapeutic uses of the reported MPs through phytochemical screening and bioassays and determining their safety through toxicological studies.
